# Abscess after Appendicitis

**Published:** 1901-03-16

**Authors:** 


### ABSCESS AFTER APPENDICITIS.

There is no doubt that many people struggle through an attack of appendicitis, sometimes without the disease being discovered, often without it being operated on. It is well then to bear in mind that what seems like convalescence may be in reality a condition full of danger and liable at any moment to land the patient in the direst peril; in other words, a comparatively quiet abscess may have formed which until the occurrence of accidental rupture may give rise to but few symptoms suggestive of its presence. Mr. Rutherford Morison,[1] of Newcastle-on-Tyne, says that, whatever controversies may still exist as to the treatment of appendicitis, all authorities are agreed that the presence of pus makes operation imperative. Yet the ordinary signs of pus formation may be absent. A rigor or pus temperature, redness and œdema in the right iliac region, or the presence of deep fluctuation, if found, are all suggestive of the presence of pus; but these ordinary signs of pus formation are so exceptionally present that if a diagnosis is to be based on them it will rarely be made. It is a fact, says Mr. Morison, and it is of overwhelming importance to know it, that all these signs may be wanting, and that with a large and dangerous abscess the patient may appear to be convalescent. The two essentials for diagnosis are:—(1) The history; that of a sudden severe attack of abdominal pain gradually becoming localised in the right iliac fossa, attended by vomiting and fever and followed by an abdominal illness; and (2) the discovery of a definite tender tumour, with usually rigidity of the abdominal muscles in the neighbourhood of the appendix. The diagnosis of appendicular abscess made upon these grounds will less often be wrong, he says, than any other in abdominal surgery, and every such case should be operated upon. Many cases are given illustrating this point and the different methods of operating according to the direction taken by the abscess. The special operation which he describes is one with a large incision planned throughout with the object of having everything in full view. The drain is left quite at the posterior angle of the wound where in fact it is dependent. Needless to say with so large a wound great care in suturing is insisted on.

[1] Lancet, Feb. 23.

